# Efficacy of polyglucosamine for weight loss—confirmed in a randomized double-blind, placebo-controlled clinical investigation

**DOI:** 10.1186/s40608-015-0053-5

**Published:** 2015-06-10

**Authors:** Karina Pokhis, Norman Bitterlich, Umberto Cornelli, Giuseppina Cassano

**Affiliations:** Salztal Klinik GmbH, Parkstrasse 18, D-63628 Bad Soden-Salmünster, Germany; Medizin & Service GmbH, Abt. Biostatistik, Boettcherstr. 10, D-09117 Chemnitz, Germany; Loyola University, School of Medicine—Chicago, 2160 South Fist Avenue, Maywood, IL USA; MAP center, Via Rossini, Rende, CS 87036 Italy

**Keywords:** Obesity, Body weight, Overweight, Weight loss, Polyglucosamine, Caloric restriction, Physical activity

## Abstract

**Background:**

The purpose of this clinical study was to ascertain whether low molecular weight chitosan polyglucosamine is able to produce significantly better weight loss than placebo.

**Method:**

115 participants were included in the study. We used a two-center randomized, double blind, placebo-controlled design. The participants followed a standard treatment (ST), which included the combination of a low-calorie diet achieved through creating a daily calorie deficit (500 cal) and an increased daily physical activity (7 MET-h/week). They were randomized to receive standard treatment plus placebo (ST + PL) or standard treatment plus polyglucosamine (ST + PG), respectively. Participants were instructed to take 2 × 2 tablets before the two meals containing the highest fat content for at least 24 weeks. Body weight, BMI, waist circumference and the time needed for a 5 % body weight reduction (5R) were taken as main variables.

**Results:**

The average weight loss over a period of 25 weeks in the ITT population was 5.8 ± 4.09 kg in the ST + PG group versus 4.0 ± 2.94 kg in the ST + PL (pU = 0.023; pt = 0.010). After 25 weeks, 34 participants achieved 5R in the ST + PG group (64.1 %) compared to only 23 participants in the ST + PL group (42.6 %) (ITT) (p Fisher = 0.033). Weight loss through hypo-caloric diets have been found to be effective. The additional effect of PG in combination with standard treatment is able to produce significantly better weight loss than placebo.

**Conclusions:**

Participants treated with ST + PG showed a significant amount of weight loss, an additional 1.8 kg, compared to controls treated with ST + PL.

**Trial registration:**

Trial Registration at ClinicalTrials.gov: NCT02410785 Registered 07 April 2015

## Background

The prevalence of obesity in European countries has trebled in the last 20 years [1] together with body weight increase. Both events have a tremendous impact on quality of life and are associated with numerous comorbidities such as cardiovascular diseases, diabetes mellitus and certain cancers.

The financial implication of these conditions is a cause for concern since both direct and indirect costs of illness, care and rehabilitation services will only grow larger over time. Measures must be taken to reverse this upward trend and as yet, no gold standard has been established for the treatment of overweight and obesity [2].

A guideline enabling practitioners and patients to make decisions about appropriate treatment and healthcare for specific clinical conditions as well as taking into account their personal circumstances, has been compiled by different professional associations in Europe as well as in Germany [3].

Additionally, medicines granted the marketing authorisation by the European Medicine Agency (EMA) but have failed to meet the expectations in their practical application will be withdrawn from the market. As a result of the increased risk of serious and sometimes fatal events, they recommended the suspension of marketing authorisations for sibutramin and rimonabant across the European Union [4,5]. Currently, orlistat is the only prescription medicine available on the European market.

Some food and food supplements promise rapid and effective weight loss in a relatively short period of time without having to implement dietary and lifestyle changes or additional physical activities.

However, weight management incorporating the use of a medical device generally recommends implementing healthy eating habits and making other lifestyle changes.

In order to promote compliance and enhance the effects of the standard treatment, some therapists recommend the use of the medical device polyglucosamine (PG). This product works chemo-physically by binding to fat in the intestinal lumen to form a complex partially used by colonic bacteria as fuel and the remaining mass is eliminated [6,7]. As a result, the absorption of fat is inhibited and thereby impairs the absorption of energy through dietary fat restriction.

This clinical study was conducted to assess the efficacy of PG against placebo. Patients also made changes in behavior patterns such as establishing healthy eating and exercise habits in addition to taking the investigational product.

The evidence-based guideline “Prevention and Treatment of Obesity” recommends making dietary changes in terms of consuming a mildly hypocaloric diet providing a daily energy deficit of around 500 kcal which contains approximately 30 % of fat [3].

However, the guideline in the current study was modified to include the additional intake of PG or placebo and an increased intake of dietary fat from 40–60 g per day to 60–80 g per day.

## Methods

### Study design

The study was conducted in two centers according to a randomized, double-blind, placebo-controlled design and in accordance with the requirements of the Directive 92/43/EEC and DIN EN ISO 14155-1 and in conformance with the provisions in the current version of the Declaration of Helsinki. The Federal Data Protection Act/Italian Personal Data Protection Code including other applicable laws, regulations, mandatory standards and commendations were also taken into consideration.

The patients were randomized to receive in addition to standard treatment (ST) either PG or placebo, 2 x 2 tablets before the daily meals containing the highest fat content.

The trial protocol was submitted to the relevant Ethics Committee of the State Chambers of Physicians in Frankfurt, Germany for evaluation, consideration and review and given a favorable opinion before the study began.

In Italy, the trial protocol was sent for approval to the Comune di Rende. The approval N14 was given according to art. 48 del D. Lgs n 267/2000 at 28^th^ January 2010.

The patients in Germany were recruited into the clinical trial from the surrounding area of Salztalklinik, Bad Soden-Salmünster [Center 1] and the patients in Italy were recruited during the MAP Study (Monitoraggio Alimenti e Patologie) in the center of Rende, province of Cosenza [Center 2].

### Participants

Altogether 115 participants were included in the study, 36 of whom were men and 79 were women between the ages of 21 to 75 years.

Inclusion criteria were as follows: BMI > 26 and < 45; waist circumference of more than 88 cm for women and greater than 102 cm for men.

Exclusion criteria were: pregnancy or breast-feeding; alcohol abuse, drug abuse or drug addiction; inability to fulfill the criteria of the trial protocol; cancer diseases, malignant tumors; pre-existence of chronic intestinal disease and known hypersensitivity reactions to crustaceans.

Another exclusion criterion was the use of disease modifying anti-diabetic drugs. Patients undergoing treatment for chronic cardiovascular diseases were accepted, provided no modification of the type of medication and dosage regimen were undertaken during the trial.

### Treatments

Each patient took 2 tablets of placebo or 850 mg of PG twice daily before the two meals containing the highest fat content.

PG consists of the combination of a 125–145 kD chitosan (low molecular weight chitosan or LMWC) with ascorbic acid and tartaric acid. The combination of these substances forms a linear polymer [4,5] that is defined as PG. Due to the fat binding capacity of the product, patients were instructed to take lipophilic medications of at least four hours apart. As a preventive measure, at least one meal should be taken with high-quality oils (extra virgin) and fats to provide the body with fat-soluble vitamins as well as essential fatty acids.

When the study started, weekly counseling and physical activity sessions were provided to the patients to achieve an average 6–7 MET-h/week (daily cycling, aerobic dance, Nordic walking or swimming 2–3 times a week). They also received a CD with information about instructions regarding daily physical activity.

In order to achieve an energy deficit of 500 kcal/day, all patients were advised to reduce their dietary fat intake by making changes to their diet according to the requirements and recommended guidelines for foods to be reduced or avoided.

Treatment began immediately after the enrollment and each subject received 3 packs of 48 tablets (about 36 days of treatment). They were requested to return for more product supply at the end of each month.

The adverse events or reactions experienced were asked at every visit and documented.

After the enrollment (visit 0 as t0), 16 visits were completed during the study period of about 26 weeks (from t1 to t16). The treatment started at visit t2 (baseline).

### Sample size

The number of patients that was required to justify the difference in weight loss between groups ST + PG and ST + PL was determined based on the calculation according to Cohen’s d with an effect size of 1.0. At a significance level of 5 % with a statistical power of 80 %, 18 patients were required in each group as determined by the Mann-Whitney-test according to the study protocol. Considering a dropout rate of up to 20 %, the enrollment target in each center was fixed to at least 23 cases / treatment group. All participants were informed that they would be free to discontinue the study at any time without giving any reason and without bearing any negative consequences.

### Randomization

The random process using computer-generated block randomization with a block size of four (program BiAS Version 9.0) was not performed by a study staff but an employee of Medizin & Service GmbH. It was implemented by means of an allocation list. The study medications (verum and placebo) were packaged in blisters of identical appearance by the manufacturer.

Patients were screened at visit 0 (week 0) to see if they fulfilled the inclusion and exclusion criteria and the baseline variables were taken again at the start of the treatment. Compliance was measured by counting the residual tablets. Patients not reaching at least 90 % of the compliance rate were excluded from the evaluation.

The evaluation of compliance in relation to caloric restriction and physical exercise was not possible.

### Statistical methods

The data were analyzed using both non-parametric (primary) and parametric (only explorative) statistics. The averages and standard deviations were calculated for all variables at the different times of observation. Differences in the time needed to achieve 5 % reduction of body weight (5R) were measured according to Nonparametric Survival Analysis (Kaplan-Meier) followed by the log-rank test. At each visit, the differences in 5R were measured according to Fisher’s exact test without correction for multiple testing.

Mann-Whitney-*U* test and (only exploratory) *t* test for independent data were used to calculate the differences between the variables pertaining to the two groups. The differences between baseline value and the value at a given follow-up visit were taken as variables to compare the two groups of treatment. Body weight reduction, BMI, waist circumference and the time needed to achieve 5R were taken as main variables. Due to the small sample size, the non-parametric tests were performed as exact tests.

## Results

### Exclusion from the clinical trial

Out of the 115 patients who gave their written informed consent, a total of 28 patients were excluded from the PP population due to the following reasons:

- Six patients had BMI values not in accordance with the proposed criteria when the treatment started (three patients had BMI values of less than 26 kg/m^2^ and three patients presented BMI values exceeding 45 kg/m^2^);
- Two patients attended only the inclusion visits; no second measurement values were available;

These eight patients were excluded from the ITT population, six patients with BMI values that did not meet the inclusion criteria at visit t1 and a further two patients where no efficacy data were available (discontinuation of study after visit t1. Missing data as a result of premature termination were carried forward with the last available data in the ITT population.

107 patients could be evaluated, 53 patients in group ST + PL (27 patients in Center one and 26 patients in Center two, respectively) and 54 patients in group ST + PG (28 patients in Center one and 26 patients in Center two, respectively).

20 patients did not meet the compliance requirements after randomization (ST + PG; n = 8 = 40 %; ST + PL; n = 12 = 60 %).

**Fig. 1:**
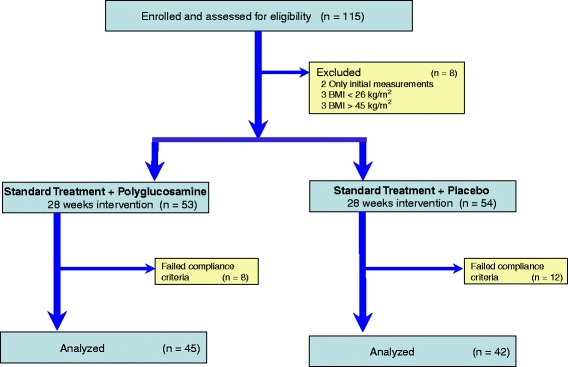
CONSORT Statement Flow Chart of the enrolment and progress of participants through the phases of the study

87 patients treated per protocol could be evaluated, 42 in group ST + PL (22 in Center one and 20 in Center two, respectively) and 45 subjects in group ST + PG (22 in Center 1 and 23 in Center two, respectively) Fig. 1.

During the first visits, a number of patients reported some discomfort such as stomach ache and meteorism. Since these symptoms were present in very few cases and in both groups, they were considered possible side effects of the early stages of making changes in diet. The complaints were mild and temporary. During the first visits following the treatment, four subjects treated with ST + PG complained of constipation, which disappeared in the following study visits after increasing water intake. Three out of the four subjects (Center 2) had a history of cholecystectomy. There were no occurrences of severe adverse events (SAE).

### Patients’ characteristics

The patients’ characteristics are shown in Table 1.

**Table 1:** Characteristics of the patients at baseline (PP): average (SD)

Variables	Measure	ST + PL group	ST + PG group	P *U* test
		(N = 42)	(N = 45)	(*t* test)
Age	Years	50.0 (8.91)	48.6 (8.67)	0.348 (0.469)
Height	Cm	167.3 (6.70)	167.7 (7.63)	0.708 (0.806)
Weight	Kg	97.9 (11.61)	98.4 (14.83)	0.884 (0.859)
Waist	Cm	108.3 (9.86)	110.4 (10.13)	0.277 (0.335)
BMI	kg/m^2^	35.0 (3.26)	35.0 (3.72)	0.758 (0.988)

There were no statistically significant differences between the groups for all the variables measured at baseline.

All the anthropometric variables decreased significantly from t2 to t16 (p < 0.001), PP and ITT in both groups. Treatment with PG significantly increased (p < 0.05) reductions in both body weight and BMI (see Table 2). Treatment in the ST + PG group was more effective in reducing abdominal circumference. However, when compared to group ST + PL, the changes were not statistically significant.

**Table 2:** Characteristics of the patients at baseline (ITT): average (SD)

Variables	Measure	ST + PL group	ST + PG group	P *U* test
		(N = 54)	(N = 53)	(*t* test)
Age	Years	50.4 (9.38)	48.8 (9.80)	0.313 (0.391)
Height	Cm	167.6 (6.24)	168.5 (8.02)	0.468 (0.519)
Weight	Kg	99.5 (11.69)	99.8 (14.95)	0.923 (0.908)
Waist	Cm	109.3 (10.14)	110.9 (10.55)	0.384 (0.431)
BMI	kg/m^2^	35.4 (3.62)	35.1 (3.73)	0.533 (0.661)

After about 9–10 weeks (corresponding to t11-t12) in the ST + PL group, all the variables on the curve caused the slope to become almost flat since no further improvement was detectable, whereas in the ST + PG group some sensitive reduction in anthropometric variables was shown.

The cut-off 5R achieved during t16 (about 25 weeks) is provided in Table 3.

**Table 3:** Values of the anthropometric measures before and at the end of the treatment (PP): average value (SD)

Times	Body weight		BMI		Waist circumf.	
	ST + PL	ST + PG	ST + PL	ST + PG	ST + PL	ST + PG
t2	97.9 (11.61)	98.4 (14.83)	35.0 (3.26)	35.0 (3.72)	108.3 (9.86)	110.4 (10.13)
t3	97.2 (11.33)	97.6 (14.55)	34.7 (3.16)	34.6 (3.75)	107.5 (9.36)	108.7 (9.73)
Changes shown as continuous variables						
t15	93.7 (12.28)	92.2 (13.68)	33.4 (3.50)	32.7 (3.46)	100.9 (9.63)	101.6 (9.55)
t16	93.6 (12.28)	91.9 (13.70)	33.4 (3.54)	32.6 (3.48)	100.9 (9.69)	101.3 (9.65)
Weeks	26.1 (2.0)	25.6 (2.0)				
Difference t16 from t2	4.3 (3.12)	6.5 (3.89)	1.6(1.18)	2.4 (1.40)	7.4 (7.66)	9.1 (6.83)
p *U* test	0.008		0.003		0.276	
p *t* test	0.005		0.004		0.293	

The 5R target was reached by 20 subjects receiving the ST + PL treatment (47.6 %) and by 32 subjects treated with ST + PG (71.1 %) Tables 4, 5, and 6.

**Table 4:** Values of the anthropometric measures before and at the end of the treatment (ITT): average value (SD)

Times	Body weight		BMI		Waist circumf.	
	ST + PL	ST + PG	ST + PL	ST + PG	ST + PL	ST + PG
t2	99.5 (11.69)	99.8 (14.95)	35.4 (3.62)	35.1 (3.73)	109.3 (10.14)	110.9 (10.55)
t3	98.6 (11.44)	99.0 (14.69)	35.1 (3.56)	34.7 (3.75)	108.6 (9.72)	109.1 (10.20)
Changes shown as continuous variables						
t15	95.6 (12.42)	94.2 (14.55)	34.0 (3.91)	33.0 (3.60)	102.7 (10.34)	102.9 (10.39)
t16	95.5 (12.43)	94.0 (14.60)	34.0 (3.94)	33.0 (3.62)	102.6 (10.40)	102.6 (10.52)
Weeks	22.9 (7.26)	23.9(5.49)				
Differencet16 from t2	4.02 (2.94)	5.83 (4.09)	1.44 (1.10)	2.14 (1.48)	6.70 (7.01)	8.34 (6.73)
p *U* test	0.023		0.009		0.266	
p *t* test	0.010		0.007		0.219

**Table 5:** Study duration and the number of 5R at the visits (PP)

Control	Days		5 %-Responder cumulative		Difference ST + PG - ST + PL Cumulative	P Fisher Test
	ST + PL	ST + PG	ST + PL	ST + PG		
t3	7.1	7.7	0	0	0	-
t4	14.4	16.6	0	1	1	1.000
Changes shown as continuous variables						
t15	149.5	152.0	17	31	14	0.010
t16	174.1	176.3.	20	32	12	0.030

**Table 6:** Study duration and the number of 5R at the visits (ITT)

Control	Days Mean (SD)		5 % Responder cumulative		Difference ST + PG - ST + PL Cumulative	P Fisher Test
	ST + PL	ST + PG	ST + PL	ST + PG		
t3	12.0 (4.4)	11.2 (4.4)	0	0	0	
t4	20.1 (5.0)	18.9 (5.0)	0	1	1	0.495
Changes shown as continuous variables						
t15	144.4 (35.7)	139.6 (39.9)	19	32	13	0.012
t16	166.6 (44.4)	161.4 (48.0)	23	34	11	0.033

**Fig. 2:**
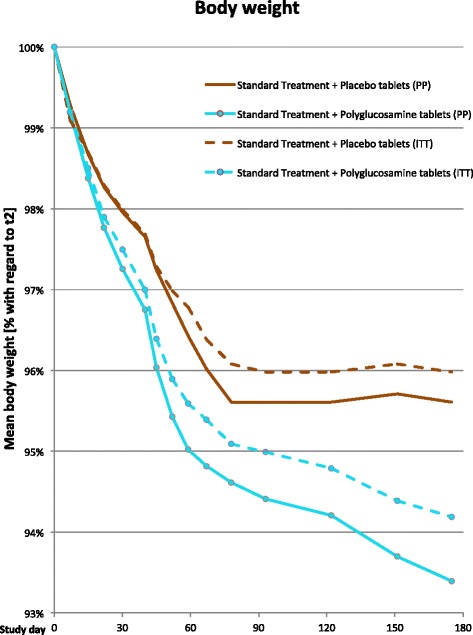
Mean body weight [% with regard to t2]. The red solid line shows the % reduction in body weight of the group (PP) receiving “standard-treatment plus placebo tablets” against weight loss and the cyan solid line in the group receiving “standard treatment plus polyglucosamine tablets” (PP), whereas the dashed lines in the respective colors denote the progress of the ITT groups

The likelihood ratio analysis indicated that a statistically significant difference (p < 0.002) was evident starting from t 8, corresponding to 44–46 days of treatment. The difference between the two groups was maintained until the end of treatment Fig. 2.

**Fig. 3:**
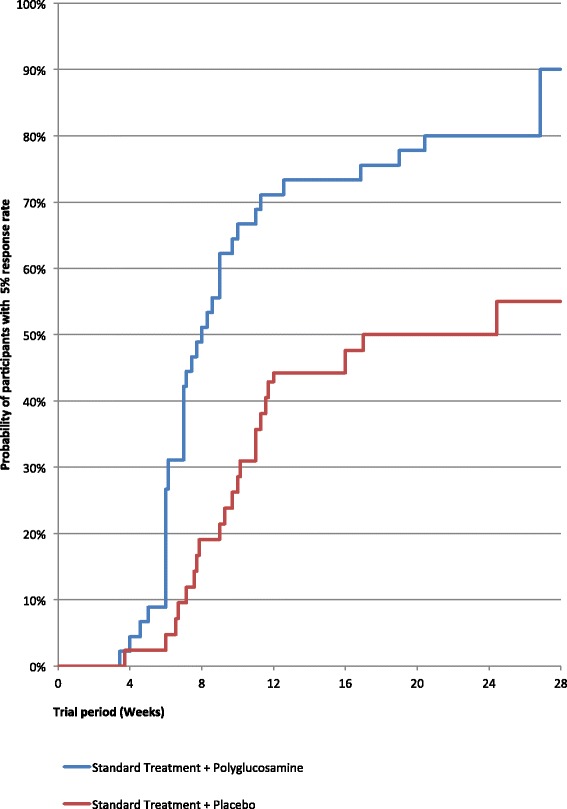
Kaplan-Meier curve estimating percentage of patients with weight loss of 5 % of initial body weight (5R)

There was a statistically significant difference between the study groups (log-rank p = 0.001) if reaching a body weight of 95 % of the initial body weight, measured at the time of the visit, was considered as the time point of achieving target weight. The median time to achieve 5R in the ST + PG group was 56 days, whereas it was 119 days in the ST + PL group. As illustrated by the Kaplan-Meier curve, patients who did not lose 5 % of their initial body weight during the study period will be recorded as censored data Fig. 3.

## Discussion

The results presented in this trial show the superiority of the concurrent treatment with PG compared to placebo. During the study period, patients received similar intervention using the standard treatment recommended by the associations [3]. The main components of this lifestyle intervention included making dietary changes according to the evidence-based guidelines (level 1 nutritional interventions) in combination with increased physical activity. The PG group had a statistically significant greater weight loss than group ST + PL.

Weight loss and mild short-term adverse effects not requiring medical attention in both groups, confirmed the success of this treatment. This outcome allows a population projection.

The possible amount of weight loss that may be achieved over a period of six months through an evidence level 1 nutritional intervention was between 3.2 and 4.3 kg. This conclusion made by the experts of the associations [3] is similar to the data obtained from other studies [8,9]. Thus, an external validity of the data and findings of this study is ensured, since the result achieved showed that in the ITT population the placebo group experienced a weight loss of 4.0 kg (CI: 3.2–4.9). The average body weight loss of an additional 1.8 kg obtained in the ST + PG group (weight loss of 5.8 kg, CI: 4.7–7.0) is therefore attributable to the mechanism of action of PG.

Compare BMI ST + PG 2.14 kg/m^2^ (CI: 1.72–2.54) versus ST + PL 1.43 kg/m^2^ (CI: 1.13 – 1.74).

Compared to the 24-week clinical trials [10,11], weight loss achieved in this study is particularly pronounced. The existing differences are explicable both in terms of types of chitosan and methodology.

There is no doubt that chitosans behave differently from the experimental and clinical point of view, depending on both the molecular weight and the combination with organic acids in their formulations.

PG is a low molecular weight chitosan (LMWC) in a fixed combination with organic acids added to avoid the polymer chain entanglement, which tends to hide the positive charges of the glucosamine moieties. The main characteristic of PG is the linearity of the polymer composition that allows a more efficient fat binding capacity [6,7]. This fat binding capacity allows lipids to reach the lower intestine where they can be used by bacteria as fuel. For this reason, the amount of fat detectable in feces will not be in the form of a steatorrhea. This observation has led some authors to claim that chitosan does not have the ability to significantly bind fat [12].

The experimental evidence that chitosans are a large family of compounds that can differ in their chemical and physical properties is an old finding [13], and that the fat binding capacity can be enhanced by the combination of chitosan with ascorbic acid is even an older one [14]. However, most of the time, both concepts have been completely ignored despite the possibility that they could be variables that may improve the clinical activity of a given compound or formula.

In clinical trials conducted in the past [15] and in recent experimental studies, the main characteristics of chitosan have not been reported [16].

In the meta-analysis conducted by Jull et al. [9] in 2009, most of the trials did not even mention the chemico-physical specifications of chitosan.

The results of the present study are similar to those of the authors treating overweight subjects with PG, in combination with light physical activity [17]. The outcome demonstrated a reduction in body weight, waist circumference and also in the risk of developing metabolic syndrome.

In the first 50 days of ST + PG treatment, 42.2 % of the subjects showed a 5 % body weight loss compared with 9.2 % in the ST + PL group.

Achieving the goal of the clinical trial within a short period of time is a good incentive for maintenance of body weight since it motivates the patients to continue with the treatment schedule and increases the patients’ confidence in their ability to manage their body weight. This aspect is important because it encourages the patients to persevere with treatment and achieve better results.

In the current clinical trial the daily fat restriction was limited to 80 g instead of the usual 60 g. Long-term fat restriction of twenty grams more per day (limiting intake to 60 g) can be frustrating.

A positive effect was the association between PG and a less severe dietary fat restriction, which helped to maintain continuation of participation in the ST + PG group.

Despite an increase in daily fat consumption between 40–60 and 60–80 g, the combined effect of ST and PG with a high fat-binding capacity led to an equally rapid and satisfactory weight loss.

One further aspect that needs to be considered in relation to the PG treatment concerns the evidence that almost 85 % of the results was obtained between 8 and 9 weeks with an evident progressive reduction of the body weight from 1 to 10 weeks. This suggests that in case PG does not produce a consistent activity (5 % body weight reduction) in the first two months of treatment, the patient has to be considered “not sensitive” and the intervention with PG will lose its cost-effectiveness ratio.

## Conclusion

The patients that obtained the 5R cut-off with PG after 9 weeks of treatment were three times more than those without (27 versus 9 subjects) and a consistent difference was maintained between the two groups until the end of the trial; 34 participants achieved 5R in the ST + PG group (64.1 %) compared to only 23 participants in the ST + PL group (42.6 %) (ITT) (p Fisher = 0.033).

## Abbreviations

%: Percent
5R: A body weight reduction of at least 5 % of the initial body weight
BDSG: German Federal Data Protection Act
BMI: Body Mass Index/kg/m^2^
cm: Centimeter (metric system)
g: Gram (metric system)
Kcal: Kilocalories
kD: Kilodalton
kg: Kilogram (metric system)
LMWC: Low molecular weight chitosan
MAP: Monitoraggio Alimenti e Patologie
MET-h: Metabolic Equivalent of Task (MET) per hour
PG: Polyglucosamine (ß-1,4-polymer of D-glucosamine and N-acetyl-D-glucosamine)
SAE: Severe Adverse Events
SD: Average (Standard Deviation)
ST: Standard treatment, recommended hypo-caloric diet, (−500 kcal) plus additional physical exercises
ST + PG: Standard treatment plus polyglucosamine-tablets
ST + PL: Standard treatment plus placebo-tablets
